# Immunohistochemical expression of P53, Ki-67, and CD34 in psoriasis and psoriasiform dermatitis

**DOI:** 10.1051/bmdcn/2019090426

**Published:** 2019-11-14

**Authors:** Mazaher Ramezani, Atefeh Shamshiri, Elisa Zavattaro, Sedigheh Khazaei, Mansour Rezaei, Rozhano Mahmoodi, Masoud Sadeghi

**Affiliations:** 1 Molecular Pathology Research Center, Imam Reza Hospital, Kermanshah University of Medical Sciences Kermanshah Iran; 2 Students Research Committee, Kermanshah University of Medical Sciences Kermanshah Iran; 3 Dermatology Unit, Department of Translational Medicine, University of Eastern Piedmont Amedeo Avogadro 28100 Novara Italy; 4 Deparment of Biostatistics, Fertility and Infertility Research Center, Kermanshah University of Medical Sciences Kermanshah Iran; 5 Medical Biology Research Center, Kermanshah University of Medical Sciences Kermanshah Iran

**Keywords:** Immunohistochemistry, Psoriasis, Dermatitis

## Abstract

Background: Psoriasis is the prime example of psoriasiform tissue pattern and should be differentiated from other psoriasiform dermatoses both clinically and histopathologically.

Aim: To evaluate immunohistochemical expression of P53, Ki-67, and CD34 in psoriasis and psoriasiform dermatitis for diagnostic purposes.

Methods: An analytical cross-sectional study was performed on the paraffin blocks of 60 psoriasis and 31 psoriasiform dermatitis patients between 2014 and 2017. The selected formalin-fixed paraffin-embedded tissues from each biopsy specimen were cut into 4-micron sections. Initial sections were stained by hematoxylin and eosin staining. Primary antihuman antibodies against P53, Ki-67, and CD34 were applied. Positive control samples for biomarkers were received from former strongly positive samples of papillary endothelial hyperplasia, high grade lymphoma, and breast ductal carcinoma for CD34, Ki-67, and P53, respectively.

Results: Out of 60 psoriasis patients, 56.7% were men, with the mean age of 36.8 years. From 31 psoriasiform patients, 45.2% were men, with the mean age of 37.5 years. Both groups were matched in terms of sex and age. The mean staining of three markers was more significant in psoriasiform dermatitis than psoriasis.

Conclusion: In spite of some other researches, the present study showed expression of P53, Ki-67, and CD34 biomarkers were significantly higher in psoriasiform dermatitis than psoriasis.

## 1. Introduction

Psoriasis is a chronic inflammatory cutaneous disorder with multifactorial, but undefined etiology which affects about 2% of the world’s population [1–4]. This disease has been characterized by dermal inflammation, hyperproliferation and incomplete differentiation of epidermal keratinocytes [5]. Histopathologically, psoriasiform dermatitis shows the epidermal hyperplasia with regular blood vessels. Psoriasis is, however, the prime example of psoriasiform dermatitis and other cutaneous disorders may disclose psoriasiform epidermal hyperplasia and create confusion in histopathologic diagnosis [6]. Differentiation between psoriasis and psorisiform dermatitis is important for diagnostic, prognostic and therapeutic purposes. This differentiation may be possible by using immunohistochemical methods [6]. P53 is a tumor suppressor gene [7, 8] including 393 amino acids [9]. Negatively, the wild-type P53 can regulate cellular proliferation *via* controlling the cell cycle [10–12]. P53 protein positivity is shown in several inflammatory cutaneous disorders including psoriasis and chronic dermatitis [13, 14]. Ki-67 as a proven marker for cell proliferation [15–17] is strongly present in psoriasis and correlates with the clinical severity of psoriasis [17]. Therefore, labeling with Ki-67 is of benefit in demonstrating proliferation in tissue, including psoriasis [18]. This marker is present in most parts of the cell cycle [15]. Lesions of psoriasis express Ki-67 more strongly than normal and non-lesional skin [19]. CD34 marker acts as adhesion [20–22] and antiadhesion [23, 24] molecules in specialized blood vessels and mast cells, respectively. This marker can have a diagnostic utility in inflammatory skin disorders [25, 26]. Herein, this study aimed to assess the differences in immunohistochemical expression of P53, Ki-67, and CD34 in psoriasis and psoriasiform dermatitis.

## 2. Material and methods

### 2.1. Patients

This analytical cross-sectional study was approved by the Ethics Committee of Kermanshah University of Medical Sciences. The patients were selected from the documented reports of pathology in which the first clinical diagnosis and biopsy-proven diagnosis were the same as psoriasis vulgaris or one of the psoriasiform dermatoses. In this study, 60 paraffin blocks of psoriasis and 31 blocks of psoriasiform dermatitis were collected from the “Special Clinic” of Kermanshah University of Medical Sciences, Kermanshah, Iran, between 2014 and 2017. Psoriasiform dermatoses were identified in specific diagnoses, but due to small number of some entities, statistical analysis mandated considering all of them under the umbrella of one term.

### 2.2. Immunohistochemical and histopathology analyses

The selected formalin-fixed paraffin-embedded tissues from each biopsy specimen were cut into 4-micron sections and then mounted on glass slides. For the first time, they were stained by hematoxylin and eosin staining. The clinical diagnosis of psoriasis and psoriasiform dermatitis was done by dermatologists who were blind to the results of histopathology. The histopathological diagnosis was made by a dermatopathologist who was blind to the clinical diagnosis. The criteria used for histopathological diagnosis of psoriasis were hyperkeratosis with confluent parakeratosis, regular acanthosis, lack of granular layer, supra papillary thinning, Munro-Sabouraud micro abscess, high mitotic rate in the epidermis, dilated tortuous capillaries in papillary dermis, and the presence of T-lymphocyte infiltration in the dermis. The selected cases had most of the criteria. The psoriasiform dermatitis cases included chronic eczema, lichen simplex chronicus, pityriasis rubra pilaris, and pityriasis rosea, and they were diagnosed according to the criteria of dermatopathology textbooks, none of which had the main criteria of psoriasis [27]. The diagnosis was confirmed by a dermatopathologist. Then, immunohistochemistry was done. Primary antihuman antibodies against P53 protein (BioGenex, clone DO7, Fremont, CA, USA), Ki-67 (DAKO, clone MIB-1, Santa Clara, CA, USA) and CD34 (BioGenex, clone QBEND/10, Fremont, CA, USA), were used, according to the manufacturer protocols. Positive control samples for biomarkers were received from former strongly positive samples of papillary endothelial hyperplasia, high grade lymphoma and breast ductal carcinoma for CD34, Ki-67, and P53, respectively. The percentage of stained cells was estimated in high power field (×400) and divided as ≥6 blood vessels in stained papillary dermis were positive for CD34 and ≥25% of epidermal cells for Ki-67 and P53 were positive. In the case of P53 and Ki-67, all the keratinocytes with stained nuclei were estimated in high power fields and an average of positivity percentage was taken on the agreement of dermatopathologist and assistant. For evaluation of CD34, all of the high power fields immediately under epidermis were screened for separated vessels with open lumen and an average of the number of separated vessels was taken on the agreement. Cut-off points of 25% for P53 and Ki-67 was considered according to literature [19, 28]. Regarding CD34 evaluation, different methods are reported in the literature, and with this in mind, we considered 6 vessels as cut-off point of positivity [29].

### 2.3. Statistical analysis

The data were analyzed by SPSS version 22 (IBM Corp., Armonk, NY, USA). The categorical and continuous data were analyzed using *Chi-*square and *t*-test, respectively. The mean (±standard deviation (SD)) was used for the continuous data and the number of participants (percentage) was used for the categorical data. The graphs were plotted by Microsoft Excel software 2010. A *p*-value (2-taild) < 0.05 was considered to be statistically significant. In all analyses, confidence interval (CI) was 95%.

## 3. Results

### 3.1. Baseline variables

Sixty cutaneous paraffin-embedded specimens taken from psoriatic lesions and 31 from psoriasiform dermatitis were collected from the same number of patients and were analyzed. Out of 60 psoriasis patients, 56.7% were men. The biopsies from psoriasiform dermatitis patients belonged to chronic eczema (77.4%), Lichen simplex chronicus (12.9%), Pityriasis rosea (6.5%), and Pityriasis rubra pilaris (3.2%). Data regarding patients and specimens are depicted in Table 1.

**Table 1:** The baseline characteristics of the patients included in psoriasis and psoriasiform dermatitis groups.

Variable	Psoriasis (n = 60)	Psoriasiform dermatitis (n = 31)	*P*-value
**Age (year)**			
Mean (±SD)	36.8 (±14.9)	37.5 (±17.7)	0.847
Range	9-78	10-78	
**Sex, n (%)**			0.206
Male	34 (56.7)	14 (45.2)	
Female	26 (43.3)	17 (54.8)	
**Diagnostic, n (%)**			
Chronic Eczema	-	24 (77.4)	-
Lichen Simplex Chronicus		4 (12.9)	
Pityriasis Rosea		2 (6.5)	
Pityriasis Rubra Pilaris		1 (3.2)	

Abbreviation: SD: Standard Deviation.

### 3.2. Immunohistochemistry staining

#### 3.2.1. P53 Staining

**Fig. 1:**
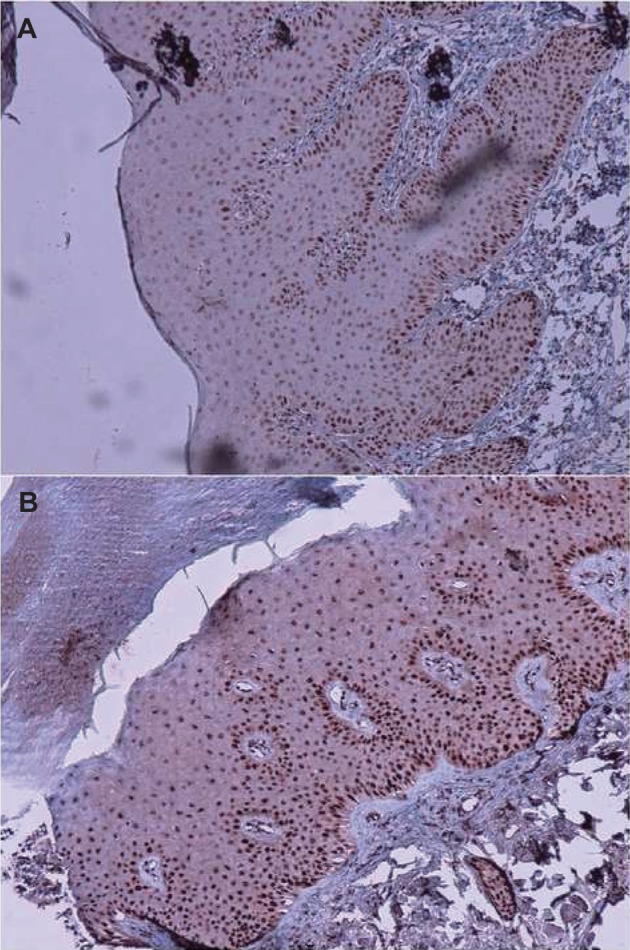
Positive staining (magnification, ×100) for P53 in (A) psoriasis and (B) lichen simplex chronicus.

Positive P53 was presented in the epidermis and viewed by brown created in the nuclei of epidermal cells. In each sample, high expression (≥25%) was considered positive (Fig 1), and positive/ negative staining results were different between psoriasiform dermatitis and psoriasis specimens (*P* = 0.006), (Table 2). In addition, there was a significant difference between psoriasis and psoriasiform dermatitis in P53 expression (mean percentage of epidermal cells stained ± SD, *P* = 0.030).

**Table 2:** Comparison of the diagnostic markers between psoriasis and psoriasiform dermatitis patients.

Variables	Psoriasis (n = 60)	Psoriasiform dermatitis (n = 31)	*p*-value
**P53**, n(%)			
Positive€	36 (60)	27 (87.1)	0.006
Mean (±SD), %*	43.1 (±32.7)	58.3 (±27.3)	0.03
**Ki-67**, n (%)			
Positive€	30 (50)	22 (71)	0.044
Mean (±SD), %*	21.6 (±10)	29 (±11.6)	0.002
**CD34**, n (%)			
Positive€€	31 (51.7)	22 (71)	0.06
Mean (±SD) **	5.7 (±2.1)	7.4 (±27.3)	0.004

€ ≥25% of epidermal cells stained; *Mean (± standard deviation (SD)) percentage of epidermal cells stained; €€ ≥6 of blood vessels stained; **Mean (±SD) number of blood vessels stained (×400); CD: cluster of differentiation.

#### 3.2.2. Ki-67 Staining

**Fig. 2:**
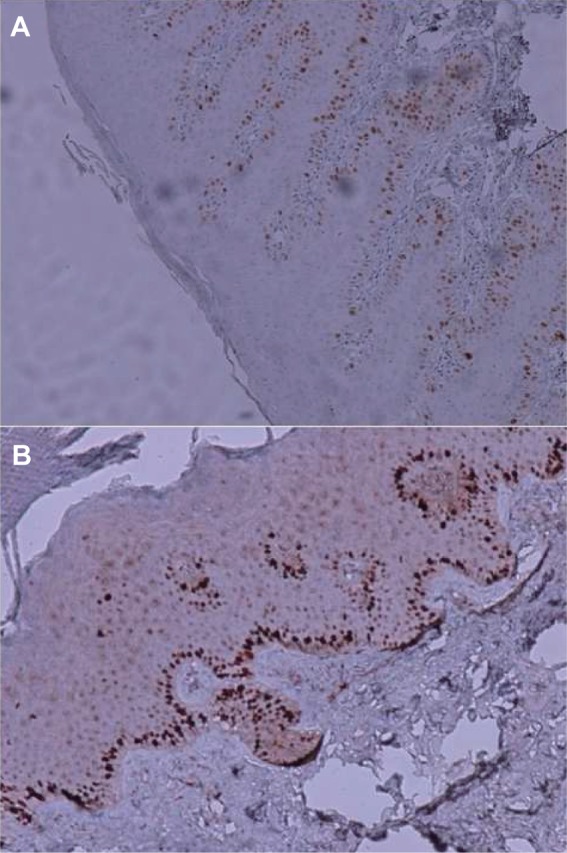
Positive staining (magnification, ×100) for Ki-67 in (A) psoriasis and (B) lichen simplex chronicus.

Positive Ki-67 was presented in the epidermis and viewed by brown created in the nuclei of epidermal cells. In each sample, high expression (≥25%) was considered positive (Fig. 2), and positive/negative staining results were different between psoriasiform dermatitis and psoriasis specimens (*P* = 0.044), (Table 2). In addition, there was a significant difference between psoriasis and psoriasiform dermatitis in Ki-67 expression (mean percentage of epidermal cells stained ± SD, *P* = 0.002).

#### 3.2.3. CD34 Staining

**Fig. 3:**
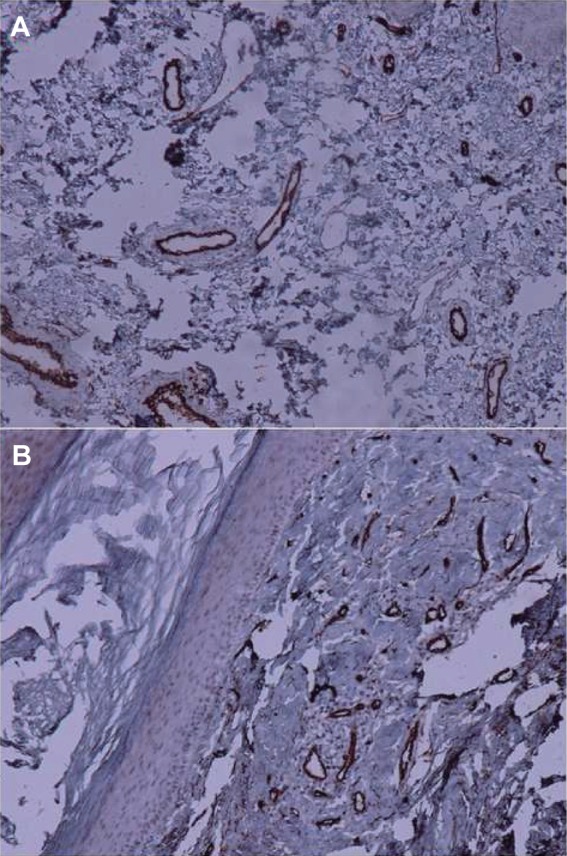
Positive staining (magnification, ×100) for CD34 in (A) psoriasis and (B) lichen simplex chronicus.

Positive CD34 was presented in the endothelial cells of vessels and viewed by brown made in the cytoplasm with or without membrane staining (Fig. 3). CD34 staining (mean count of vessels at ×400 magnification ± SD) showed a higher expression in psoriasiform dermatitis than in psoriasis specimens (*P* = 0.004), (Table 2). But there was no significant difference between psoriasis and psoriasiform dermatitis in positive/negative staining results (*P* = 0.060).

## 4. Discussion

Psoriasis has a variety of clinical features that imitate various skin conditions [30]. Histologically, psoriasis vulgaris should be distinguished from psoriasiform dermatitis, which assigns to a type of tissue pattern mimicking psoriasis, both histologically and clinically [31–34].

In the present study three immunohistochemistry markers (P53, Ki-67, and CD34) were used in cutaneous specimens collected from patients affected by psoriasis vulgaris and psoriasiform dermatitis, in order to determine which of them had a higher expression.

Tadini *et al.* demonstrated P53 expression in the cell nuclei of psoriatic skin for the first time [35]. Contrary to these discoveries, after a few years another research [36] applied similar antibodies, but did not detect P53-positive cells in the skin biopsies of psoriatic patients. A further study showed that the number of P53-positive cells was significantly higher in psoriatic lesions than non-psoriatic skin and controls [12]. Moorchung *et al.* [37] found a weak correlation between the grades of P53 immunostaining in the epidermal cells and the lesional psoriatic skin compared to the normal skin or non-lesional skin. Other authors have found that P53 is overexpressed in the keratinocytes of psoriatic epidermis [38]. A study on 30 Egyptian patients with psoriatic plaques showed that P53 nuclear staining was detected in 43.3% patients [10]. Our results showed the higher expression of P53 in psoriasiform dermatitis than psoriasis with a cut-off of ≥25% nuclear positivity in epidermal nuclei. Different results in the studies are partly related to using different methods. Psoriasis was compared with non-lesional skin and healthy controls, not psoriasiform dermatitis in one study [12]. Dermal lymphocyte and basal keratinocyte immunostaining was evaluated in another study [37], while we estimated nuclear positivity in whole epidermis. Mainly psoriasis is compared with normal and non-lesional skin and the higher expression of P53 is predictable in this comparison.

Regarding Ki-67 expression, Batinac *et al.* [39] demonstrated a prominent rise in the involved psoriatic skin than normal (17.05 *vs.* 3.65), and, simultaneously, samples with a more percentage of Ki-67 positivity demonstrated a more percentage of P53-positive cells. The study of Amin and Azim [29] showed that, expression of Ki-67 between psoriatic lesions and non-involved skin was statistically different. They also found this difference between non-involved skin and controls.

However, another study showed that Ki-67 expression was statistically higher in psoriasis than normal skin of control group.

There was no significant difference between involved and uninvolved skin of psoriatic patients [40]. An important and practical result in the study of Sezer *et al.* [6] was determination of 75% as cut-off in suprabasal/total epidermal cell count ratio for Ki-67 expression for differentiation between psoriasis and pityriasis rubra pilaris. All psoriatic patients showed positivity higher than 75%. We did not use the ratio for Ki-67 in our research, but we counted total epidermal cells and estimated epidermal cells stained for Ki-67 in whole epidermis. Although we and the Sezer group [6] both used DAKO antibodies, selecting cut-off of 25% in our research and suprabasal/total epidermal cell count ratio in their research, makes the comparison difficult. Meanwhile most of our cases were eczema than pityriasis rubra pilaris.

The study of Gupta *et al.* [41] on skin biopsies of psoriasis demonstrated higher CD34 positivity on routine microscopy and microvessel length density was significantly more in psoriasis than psoriasiform lesions. Microvessel length density was also higher in psoriasis but without a significant difference (*P* > 0.05). The results of a study by Amin and Azim [29] showed significant differences in the CD34 expression between both lesional and non-lesional skins in psoriasis and control groups. We used cut-off of ≥6 vessels/HPF in papillary dermis for CD34 biomarker, but the difference was not statistically significant (*p* = 0.060). On the other hand counting vessels without selecting a cut-off showed higher positivity in psoriasiform dermatitis than psoriasis (*p* = 0.004). Limitation of our study was making a decision for tortuous vessels. We did not use image analyzer. The numerous studies have reported different cut-offs for these markers, and they have been done on different populations; hence, this variability could also be linked to such reason. On the contrary, any differences in staining have been reported in various body sites.

## 5. Conclusions

In spite of some other researches, the present study showed a striking expression of P53, Ki-67, and CD34 biomarkers in psoriasiform dermatitis compared to psoriasis vulgaris specimens collected from Iranian patients. Future researches are needed to check these markers on more patients in different areas with different cut-offs.
